# Functional recovery of chronically critically ill patients in the first days after discharge from the intensive care unit: Feasibility of the 6-minute step test

**DOI:** 10.1371/journal.pone.0293747

**Published:** 2023-11-02

**Authors:** Viviane Roccasecca Sampaio Gaia, Eduardo Leite Vieira Costa, Wellington Pereira Yamaguti, Davi de Souza Francisco, Renata Rego Lins Fumis

**Affiliations:** 1 Hospital Sírio Libanês, São Paulo, São Paulo, Brazil; 2 Laboratório de Pneumologia LIM-09, Hospital das Clinicas HCFMUSP, Faculdade de Medicina, Universidade de Sao Paulo, Sao Paulo, SP, Brazil; 3 Research and Education Institute, Hospital Sírio-Libanes, Sao Paulo, São Paulo, Brazil; Stanford University School of Medicine, UNITED STATES

## Abstract

**Background:**

Survivors of chronic critical illness often experience weakness and functional dependence to various degrees after their intensive care unit (ICU) stay. Evaluating their functional status with the traditional six-minute walk test is challenging due to space constraints or patient intolerance.

**Objective:**

Our aim was to evaluate the feasibility of using the six-minute step test (6MST) as a measure of functional capacity in chronically critically ill patients early after ICU discharge.

**Methods:**

This prospective study was undertaken in a private Brazilian hospital. From July 2019 to July 2020, all chronically critically ill patients were asked to participate 48 hours after ICU discharge. On the day of study inclusion and a week later, those who consented underwent functional assessment comprised of the 6MST, peripheral muscle strength using handgrip strength (HGS), and mobility using the ICU mobility scale (IMS).

**Results:**

A total of 40 patients were included. The 6MST was feasible in 40% on the first evaluation and 57% on the second. The median 6MST was 0 [0–5] on the first evaluation and 3.5 [0–7.75] on the second (P = 0.005). The median HGS increased from 11.50 [9.25–18] on the first evaluation to 14.5 [10–20] on the second (P = 0.006). The median IMS was 4.5 [3.25–7] on the first evaluation and 6 [3.25–7] on the second (P<0.001). Despite the significant improvement, all parameters measured remained well below normal.

**Conclusion:**

The 6MST was a feasible measure of functional capacity in chronically critically ill patients early after ICU discharge. Patients had functional capacity well below predicted values.

## Introduction

Chronic critical illness (CCI) is characterized by a long stay in the intensive care unit (ICU) in need of advanced life support and affects a growing population of ICU survivors. A new working definition of CCI was proposed in the ProVent study as patients who spent at least eight days in the ICU and who presented one of five eligible clinical conditions: 1) mechanical ventilation for at least 96 hours in a single episode; 2) tracheostomy; 3) sepsis or other severe infections; 4) extensive wounds; 5) stroke or and traumatic brain injury [1]. After discharge from the ICU, these patients continue to demand a high level of care [2] due to significant muscle weakness and physical disability, which can persist for up to five years or more [3, 4]. Muscle weakness is an important complication in chronically critically ill patients, especially in those who survived the most severe forms of acute illness. Another contributing factor to the profound physical and functional deficits is the reduced mobility associated with mechanical ventilation [1, 5–7].

The long-term prognosis of CCI is grim. Only 10% of patients are alive and functionally independent at home one year after discharge [5, 8–10]. These patients worsen clinically and have more readmissions with worsening functionality and quality of life [11, 12]. Being cared for in a private hospital was associated with a lower risk of functional dependence at 90 days compared to a public hospital despite equivalent baseline comorbidities and acute disease [11], a finding suggestive of the existence of modifiable risk factors. The Society of Critical Care Medicine (SCCM) recommends improving the continuity of treatment for ICU survivors, involving risk assessment and comprehensive documentation during all recovery phases [13].

One test recommended for this continuous documentation of the functional status is the 6-minute walk test (6MWT), a test easy to apply in outpatients [14]. However, in many hospital settings, applying the 6MWT might not be feasible due to space constraints, limited personal resources, or due to patient intolerance. We hypothesized that using the 6-minute step test (6MST) [15], a submaximal test that requires little space to assess exercise capacity, would be feasible in patients with CCI.

Hence, the main objective of this study was to make these assessments in patients in the immediate post-ICU (48 hours) and on the seventh day after the first evaluation, together with measurements of muscle strength and mobility.

## Materials and methods

### Study design and ethical aspects

This prospective cohort study was conducted in Hospital Sírio-Libanês, a private tertiary hospital with a 63-bed ICU in São Paulo, Brazil.

### Participants

Patients discharged from the ICU were eligible if they fulfilled the criteria of CCI according to the Provent Study working definition: at least eight days in the ICU with one of the five eligible clinical conditions (mechanical ventilation for at least 96 hours in a single episode; tracheostomy; sepsis or other severe infection; extensive wounds; stroke or traumatic brain injury) [1]. The exclusion criteria for the study were: life expectancy ≤ 48 hours; functional dependence prior to hospitalization (defined as a Katz score <5, a score that ranges from 0 to 6, with a lower score indicating greater frailty and dependence on others for daily activities); patients transferred to the ward and not to the step-down unit; patients with orthopedic, neurological, or hemodynamic contraindications to performing the functional tests. All patients underwent physical therapy according to the institutional protocol.

### Outcomes

The primary outcome was the 6MST. Secondary outcomes included peripheral muscle strength using handgrip strength and mobility assessed using the ICU mobility scale. The assessments were conducted by a sole evaluator, V.R.S.G., a highly experienced physiotherapist specialized in critical care and patient rehabilitation. Furthermore, for each patient, the following information was collected: age, gender, marital status, level of education, the reason for ICU admission, comorbidities, dementia, Katz score, Simplified Acute Physiology Score 3 (SAPS 3), Glasgow Coma Scale score, Sequential Organ Failure Assessment (SOFA) score, acute physiology and chronic health evaluation (APACHE II), ICU length of stay (LOS), mechanical ventilation requirement, use of vasopressors, renal replacement therapy (RRT), blood transfusion, type of nutrition, skin integrity (bedsores), delirium judged by the confusion assessment method (CAM-ICU), and final outcome (discharge or death).

#### 6MST

The 6MST followed the same principles of the American Thoracic Society for the 6MWT [14]. A 15-cm high step was placed in the corner of the room to ensure that the step did not move during the test. Patients were allowed to keep their arms supported to perform the test. Before the test, blood pressure (BP), respiratory rate (RR), heart rate (HR), and peripheral oxygen saturation (SpO_2_) were measured. The modified Borg scale was used to assess dyspnea and lower limb fatigue at the beginning, at the end of the test, and in the second minute of recovery. BP, RR, HR, and SpO_2_ were measured again at the end of the test and in the second minute of recovery. Predicted values for this population were obtained based on the equation previously proposed [16].

**Handgrip strength (HGS)** was evaluated using a manual hydraulic dynamometer (model SH 5001, Saehan brand) according to the protocol recommended by the American Society of Hand Therapists (ASHT) [17]. Patients were instructed to remain seated in a chair, with the shoulders in a neutral position, one of the hands resting on the thigh, the contralateral elbow flexed at 90 degrees, and the forearm in neutral rotation. The dynamometer footprint was individually adjusted according to the size of the hands, with the rod closest to the dynamometer body positioned under the second phalanges of the index, middle, and annular fingers. The measurements were performed for the dominant hand. The rest period between the measurements was one minute. The best mark among three acceptable evaluations was considered the measure of handgrip strength.

**The ICU mobility scale (IMS)** is formatted on an ordinal scale of 11 points based on the maximum degree of activity the patient performs according to mobility milestones. Level “0” corresponds to passive mobilization in bed, and level “10” indicates the patients’ ability to walk independently [18, 19]. Only patients who score 9 or 10 are able to execute the 6MWT test whereas those who score at least 4 can execute the 6MST.

### Statistical analysis

The statistical software GraphPad Prism 8 and R (R Foundation for Statistical Computing Platform, version 4.2.1) were used for the data analyses. Data were presented as mean and standard deviation or median and interquartile range (25–75%) as appropriate. Paired t tests or Wilcoxon signed rank tests were used to compare the first and second evaluations as indicated. Because of the COVID-19 pandemic, we included a *post hoc* analysis of COVID-19 vs. non-COVID-19 patients. For this comparison, the independent t-test for parametric data or the Mann-Whitney U tests for non-parametric data were used. Pearson’s and Spearman’s tests were used to analyze the correlations according to the normality of the data. The magnitudes of the correlations were considered low (0.26 to 0.49), moderate (0.50 to 0.69), high (0.70 to 0.89), and very high (0.90 to 1.00) [20]. The level of significance adopted was 5%.

## Results

**Fig 1:**
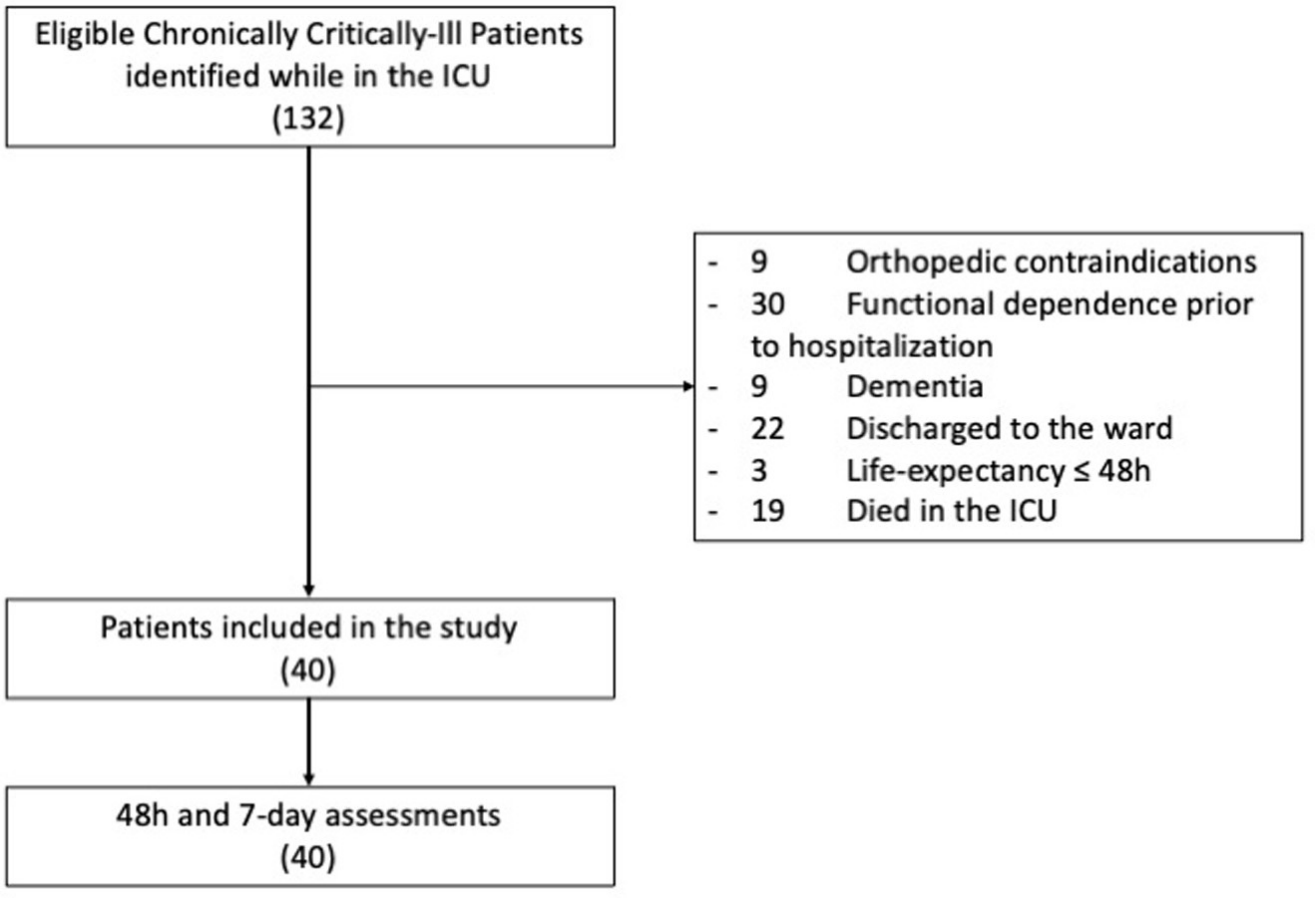
Flowchart. Screening and Assessments.

From July 2019 to July 2020, 132 patients eligible for the study were screened in the adult ICU, and 40 patients were included. Reasons for non-inclusion are outlined in Fig 1.

### Patient characteristics

Demographic and clinical characteristics are described in Table 1. Sepsis and invasive mechanical ventilation for four or more consecutive days were the most prevalent eligibility criteria for CCI.

**Table 1:** Demographic and clinical characteristics of sample.

Characteristics of sample	n = 40
Age (years)	69.8 ± 12.8
Female (n, %)	8 (20%)
Marital status, married (n, %)	34 (85%)
Education level, higher education (n, %)	35 (87.5%)
SAPS 3 (points)	57.2 ± 3.6
APACHE 2 (points)	19.7 ± 7.2
SOFA (points)	6.4 ± 3.2
Charlson (points)	0.0 [0.0–2.8]
Katz (points)	6.0 [5.0–6.0]
Covid-19 (n, %)	23 (57.5%)
Sepsis (n, %)	39 (97.5%)
Cancer (n, %)	10 (25%)
Pneumonia (n, %)	8 (20%)
Delirium (n, %)	27 (67.5%)
ICU length of stay (days)	14.0 [10.0–27.3]
Hospital death (n, %)	7 (17.5%)
Mechanical ventilation (days)	6.5 [3.0–18.8]
Mechanical ventilation ≥ 96 hours (n, %)	29 (72.5%)
Vasopressors (n, %)	36 (90%)
Renal replacement therapy (n, %)	10 (25%)
Parenteral nutrition (n, %)	7 (17.5%)
Blood transfusion (n, %)	16 (40%)

SAPS 3: Simplified Acute Physiology Score 3; APACHE 2: Acute Physiology and Chronic Health Disease Classification System 2; SOFA: Sequential Organ Failure Assessment; Covid-19: Coronavirus Disease 2019; ICU: Intensive Care Unit.

### First assessment: 48 hours after ICU discharge

**Fig 2:**
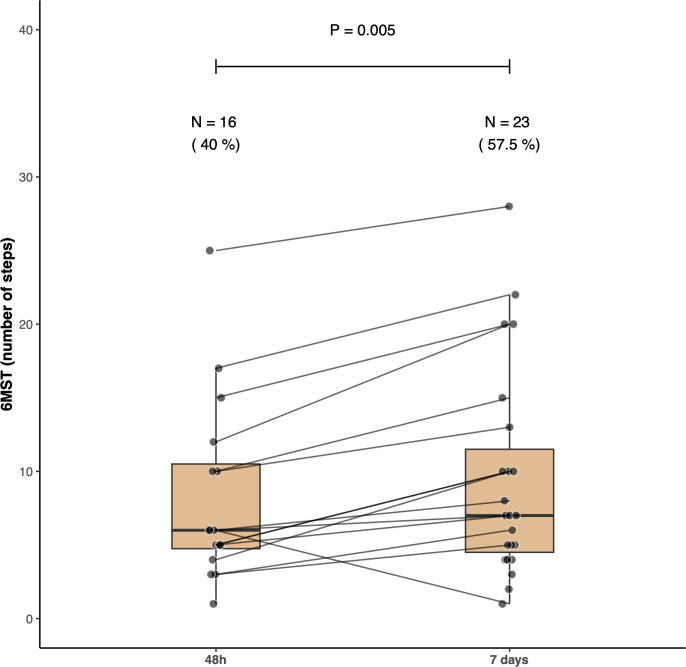
Six-minute step test (6MST). Boxplots indicate the 6MST performed 48 hours and 7 days post intensive care unit discharge to the step-down unit. Closed circles represent each patient with lines connecting the same patient in the two timepoints. Note that the number of patients able to perform the test improved from 48 hours to 7 days. Additionally, their performance on the test improved among those able to perform both tests.

**Fig 3:**
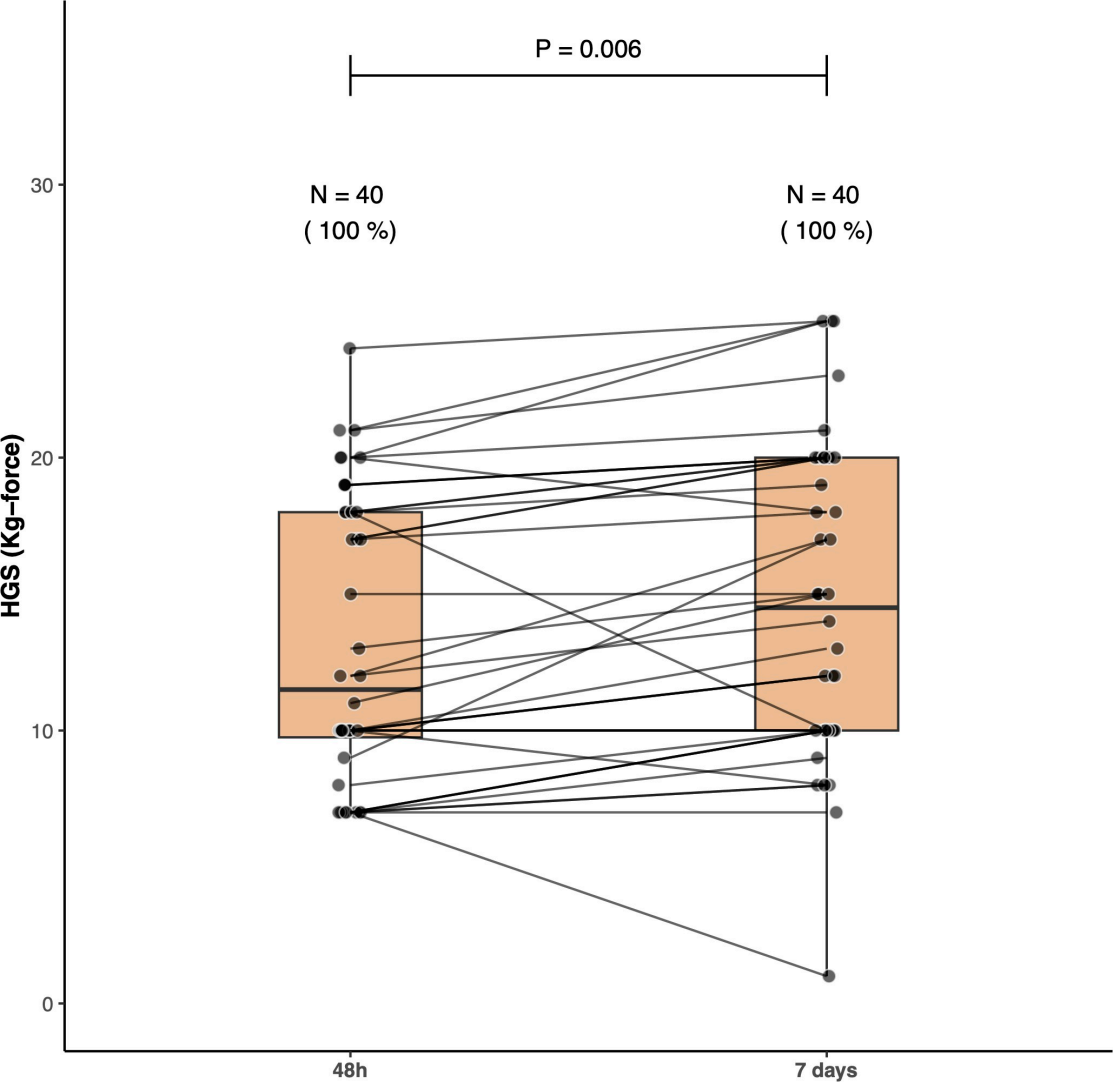
Handgrip strength (HGS). Boxplots demonstrate the HGS measurements taken at two time points—48 hours and 7 days—following patients’ discharge from the intensive care unit (ICU) to the step-down unit. Each patient is represented by a closed circle, with lines connecting their data points at the two timepoints. All patients successfully completed the HGS test at both timepoints (48 hours and 7 days). Notably, there was a significant increase in patients’ handgrip strength within the first week after being discharged from the ICU.

**Fig 4:**
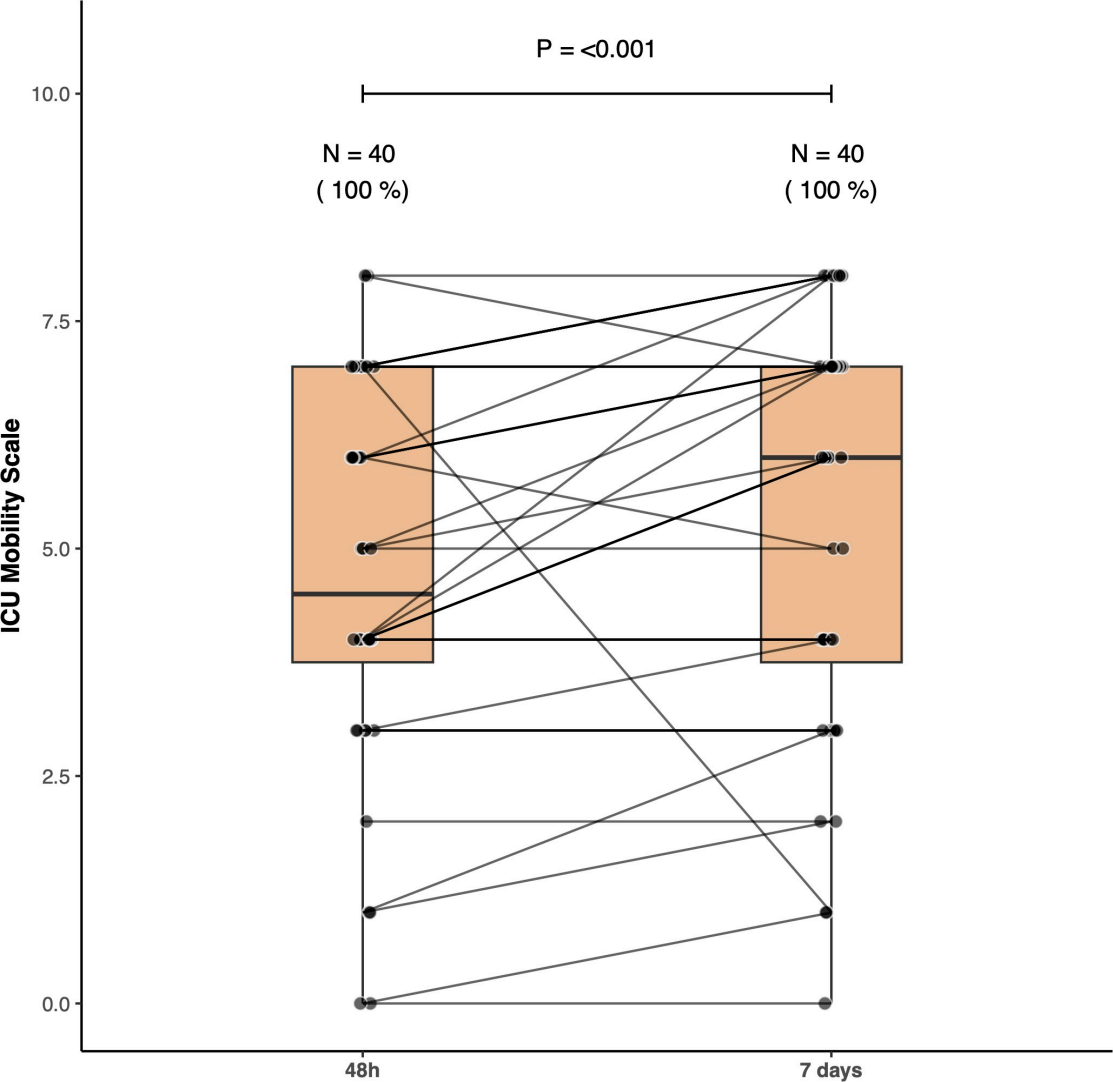
Intensive care unit mobility scale (IMS). Boxplots show the IMS performed 48 hours and 7 days post intensive care unit discharge to the step-down unit. This ordinal scale of 11 points is based on the maximum degree of activity the patient is able to perform. Level “0” corresponds to passive mobilization in bed, and level “10” indicates the patients’ ability to walk independently. Note that patients’ mobility improved over time. Closed circles represent each patient with lines connecting the same patient in the two timepoints.

Sixteen patients (40%; 95% CI 25–57%) were able to perform the 6MST. The median 6MST was 0 [0 – 5] steps corresponding to 0 [0–3.45] % of the predicted value (Fig 2). The HGS resulted in a median of 11.50 [9.25–18] Kg-force (Fig 3) or 32.56 [23.17–45.15] % of the predicted value. The IMS patients’ scores had a median value of 4.5 [3.25–7] (Fig 4). None of the patients achieved a score of 9 or higher. The 6MST had a high correlation with the HGS (r = 0.79, p <0.001) and with the IMS (r = 0.80, p <0.001). We found a low correlation with the days of mechanical ventilation (r = - 0.38, p = 0.01).

### Second assessment: 7 days later

More patients (23, 57.5%, 95% CI 41–73%) were able to perform the 6MST. Among the patients who completed the 48-hour test, only one was unable to perform the test on the seventh day. Eight patients who were unable to perform the 6MST at 48 hours improved enough to complete the test on the seventh day. Among the 15 patients who completed both tests, the median increased from 6 [5 – 11] to 10 [7 – 18] steps in the second assessment (Fig 2, p = 0.005). Despite a significant improvement, they remained well below the predicted values of 128.8 ± 22 steps. The median HGS increased to 14.50 [10–20] (Fig 3, p = 0.006 for the comparison with the 48-hour test), although the patients’ performance was still below the predicted values (median of 40.64% [35.34–44.80]). The median IMS also increased to 6 [3.75–7] (Fig 4, p <0.001 for the comparison with the 48-hour assessment) but still no patient obtained a score of 9 or higher.

In the second assessment, 6MST remained highly correlated with the HGS (r = 0.83, p <0.001) and with the IMS (r = 0.9, p <0.001).

Patients with COVID-19 remained on mechanical ventilation longer. They had similar performance on the 6MST as compared to non-COVID-19 patients, but a worse performance on the HGS and IMS within 48 hours after ICU discharge (Table 2).

**Table 2:** Comparison of demographic, clinical and functional variables between patients with and without Covid-19.

Characteristics	Covid-19 (n = 23)	Without Covid-19 (n = 17)	P
Demographic variables			
Age (years)	68.90 ± 10.10	70.90 ± 16.00	0.27
**Clinical variables**			
SAPS 3 (points)	53.70 ± 10.30	61.90 ± 16.10	0.05
APACHE 2 (points)	16.90 ± 5.40	23.40 ± 7.90	<0.001*
SOFA (points)	5.47 ± 3.14	7.42 ± 2.98	0.08
Charlson (points)	1.00 [0.00–2.00]	2.00 [0.50–6.00]	0.02*
Katz (points)	6.00 [5.00–6.00]	6.00 [5.50–6.00]	0.33
Mechanical ventilation (days)	10.00 [5.00–31.00]	3.00 [1.00–10.50]	<0.001*
ICU length of stay (days)	22.04 ± 13.76	14.71 ± 8.68	0.05
Hospital length of stay (days)	37.00 [23.00–76.00]	39.00 [26.50–68.50]	0.86
**Functional variables**			
HGS 48 hours (Kgf)	10.00 [7.00–17.00]	18.00 [10.00–19.50]	0.01*
% Predicted value	28.86 ± 10.14	44.10 ± 18.74	0.002*
HGS 7 days (Kgf)	13.6 ± 6.0	16.2 ± 5.4	0.16
% Predicted value	33.69 ± 13.13	45.36 ± 18.16	0.02*
6MST 48 hours (step)	0.00 [0.00–4.00]	3.00 [0.00–6.00]	0.09
% Predicted value	0.00 [0.00–3.07]	2.55 [0.00–4.27]	0.07
6MST 7 days (step)	0.00 [0.00–10.00]	5.00 [0.00–7.50]	0.37
% Predicted value	0.00 [0.00–6.75]	3.18 [0.00–5.57]	0.38
IMS 48 hours (points)	4.00 [3.00–4.00]	6.00 [5.00–7.00]	<0.001*
IMS 7 days (points)	4.87 ± 2.36	6.00 ± 2.16	0.10

Covid-19: Coronavirus Disease 2019; M: Male; F: Female; SAPS 3: Simplified Acute Physiology Score 3; APACHE 2: Acute Physiology and Chronic Health Disease Classification System 2; SOFA: Sequential Organ Failure Assessment; ICU: Intensive Care Unit; HGS: Handgrip Strength; 6MST: Six-Minute Step Test; IMS: Intensive Care Unite Mobility Scale.

*p<0.005.

## Discussion

We found that chronically critically ill patients fared worse than predicted in the 6MST and the HGS 48 hours after their discharge from the ICU. The 6MST was feasible in over half of patients, who would not otherwise have been able to take the 6MWT considering their score on the IMS scale. Despite their significant improvement, patients remained well below predicted values seven days later.

Our study revealed that patients’ mobility increased by 30% from 48 hours to 7 days, as evidenced by their Individualized Mobility Scale (IMS) scores. Notably, no patient achieved a score of 9 or higher in either evaluation. This suggests that all of the patients would have been unable to perform the 6-Minute Walk Test (6MWT) test within a week of ICU discharge [14]. However, 40% of patients were able to perform the 6-Minute Step Test (6MST) at 48 hours, and 57% were able to perform it at 7 days, indicating an improved feasibility of this test for early assessment of functional status while patients are still hospitalized. Those patients who were able to perform the test showed improved performance at 7 days, a clinical course that would not have been captured by the 6MWT. Although better, their performance at 7 days was still far from the predicted values.

Conversely, all patients performed the handgrip test in both evaluations. In patients who were able to perform both the HGS and the 6MST, there was a strong correlation between the tests, suggesting the importance of this test especially in patients unable to perform the 6MST. We found that patients were weak with handgrip strength below 50% of predicted, despite the improvement along the first week. It is well established that handgrip strength is an indicator of overall muscle function [21] and also an independent predictor for poor clinical outcomes in chronic critical patients [22].

Our results demonstrate the feasibility of the evaluation of musculoskeletal changes and mobility early after ICU discharge. Patients’ mobility improved within one week after ICU discharge from orthostatism to stationary gait, which still characterizes functional dependence. These findings are in accordance with previous studies of chronically critically ill patients [5] and justify the limited application of functional tests such as the 6MWT test early after ICU discharge in these chronically critically ill patients.

In this challenging scenario, we have demonstrated that the 6MST and HGS can be useful tools for tracking the early clinical course of these patients. These tests can be valuable, for example, to measure response to interventions aimed at improving physical function after ICU discharge. Future research endeavors should prioritize validating these tools using long-term outcome data and quality-of-life metrics, with the aim of establishing correlations between early functional assessment and these patient-centered outcomes.

Our study has some limitations. First, it was carried out in a single center with a small number of patients, factors that could compromise generalizability. Second, only patients discharged from the ICU to the step-down unit were selected, excluding those transferred to the wards, likely with less severe disease. Finally, we employed a step height of 15 cm, in contrast to the common practice of 20 cm in most studies.

## Conclusion

Chronically critically ill patients had profound physical impairment and partially recovered after a week. Early monitoring of functional status including the 6MST embedded in a structured rehabilitation program proved feasible and safe for most of these patients soon after ICU discharge.

## Supporting information

S1 Checklist *PLOS ONE* clinical studies checklist. (DOCX)
